# Combining Gene Transfer and Nonhuman Primates to Better Understand and Treat Parkinson’s Disease

**DOI:** 10.3389/fnmol.2019.00010

**Published:** 2019-02-11

**Authors:** Christelle Lasbleiz, Nadine Mestre-Francés, Gina Devau, Maria-Rosario Luquin, Liliane Tenenbaum, Eric J. Kremer, Jean-Michel Verdier

**Affiliations:** ^1^MMDN, University of Montpellier, EPHE, INSERM, U1198, PSL University, Montpellier, France; ^2^Department of Neurology, Clinica Universidad de Navarra, Pamplona, Spain; ^3^Laboratory of Molecular Neurotherapies and NeuroModulation, Clinical Neuroscience Department, Lausanne University Hospital, Lausanne, Switzerland; ^4^Institut de Génétique Moléculaire de Montpellier, University of Montpellier, CNRS, Montpellier, France

**Keywords:** Parkinson’s disease, primate, CAV vectors, gene transfer, dopaminergic neurons

## Abstract

Parkinson’s disease (PD) is a progressive CNS disorder that is primarily associated with impaired movement. PD develops over decades and is linked to the gradual loss of dopamine delivery to the striatum, *via* the loss of dopaminergic (DA) neurons in the substantia nigra pars compacta (SNpc). While the administration of L-dopa and deep brain stimulation are potent therapies, their costs, side effects and gradual loss of efficacy underlines the need to develop other approaches. Unfortunately, the lack of pertinent animal models that reproduce DA neuron loss and behavior deficits—in a timeline that mimics PD progression—has hindered the identification of alternative therapies. A complementary approach to transgenic animals is the use of nonhuman primates (NHPs) combined with the overexpression of disease-related genes using viral vectors. This approach may induce phenotypes that are not influenced by developmental compensation mechanisms, and that take into account the personality of animals. In this review article, we discuss the combination of gene transfer and NHPs to develop “genetic” models of PD that are suitable for testing therapeutic approaches.

## Introduction

**Figure 1:**
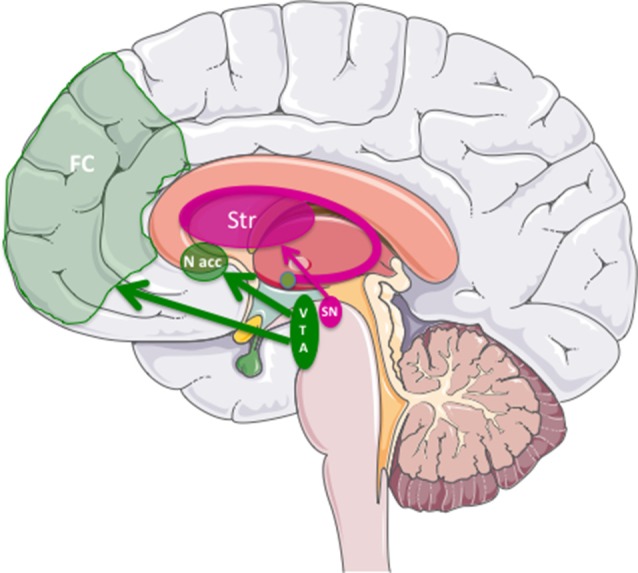
The three major dopaminergic (DA) pathways in the brain linked to Parkinson’s Disease (PD). The nigrostriatal pathway was DA cells from substantia nigra pars compacta (SNpc) project into the striatum (in dark pink). The mesolimbic/mesocortical pathway, which corresponds to the projection of the midbrain ventral tegmental area (VTA) to the *nucleus accumbens* (N. Acc) in the limbic areas, and to the frontal cortex (FC), respectively.

Parkinson’s disease (PD) is a disorder of the CNS primarily due to the degeneration of nigro-striatal dopaminergic (DA) neurons. Early symptoms are movement-related, including shaking, rigidity, slowness of movement, postural instability and difficulty with walking. Collectively, these symptoms are called “parkinsonism”. However, non-motor symptoms, such as depression and apathy, which are attributed to the degeneration of the mesolimbic mesocortical DA pathway [neurons of the ventral tegmental area (VTA) projecting to the *nucleus accumbens*, Lewis et al., 2003; Carriere et al., 2014; Dujardin and Lopes, 2014; Dujardin et al., 2014], frequently appear before the motor symptoms (Figure 1). In addition to fine-tuning of motor function, these pathways are also involved in reward (motivation), pleasure, compulsion and perseveration. Later, sensory, sleep, emotional problems, depression and dementia may arise in the late stages. Regarding the latter, the degeneration of non-DA neurons (e.g., serotoninergic) are thought to contribute to depression (Tan et al., 2011). These early neuropsychiatric manifestations often precede motor symptoms, which appear when approximately 70% of the substantia nigra pars compacta (SNpc) DA neurons are lost or are unable to deliver dopamine to the striatum. This implies that PD is, in part, an axonopathy (O’Keeffe and Sullivan, 2018). Finally, cognitive symptoms like dementia and hallucinations tend to appear in the late phases of the disease and are related to perturbation of the mesocortial pathway, which connects the VTA to the prefrontal cortex. Eventually, deficits in the noradrenergic, serotoninergic and acetylcholinergic systems also appear. Clearly, early diagnosis will be of utmost importance for disease prevention/reversal.

A handful of genes are involved in monogenic (recessive or dominant) forms of PD. Together these genes account for around 30% of familial forms and 3%–5% of sporadic cases (Klein and Westenberger, 2012). Among these PD-related genes, α-synuclein (SNCA) and leucine-rich repeat kinase 2 (*LRRK2* or *PARK8*) or are the best characterized because over-expression and/or mutations in *SNCA* and *LRRK2* are responsible for autosomal-dominant PD forms. A mutation in *SCNA* that causes an A53T change was identified in four families (Polymeropoulos et al., 1997). Since then, other mutations, duplication and triplication of this gene have been linked to PD (Deng and Yuan, 2014). LRRK2^G2019S^ is the most common mutation in familial and sporadic PD. LRRK2 mutations are also found in sporadic cases further supporting the prominent role of this gene in PD aetiology. Finally, disease evolution of patients with LRRK2 mutations, including the accumulation of Lewy bodies, are clinically indistinguishable from those with idiopathic PD (Gasser, 2009). Familial forms of PD are slowly providing clues to underlying mechanisms of neurodegeneration.

While some drugs have markedly improved parkinsonism, their efficacy often declines as PD progresses. To date, there are no long-term disease-modifying treatments available for the 10 million people worldwide suffering from PD. Therefore, using pertinent models that allow the scientific community to develop new approaches are of utmost importance to combat PD.

## Acute and Chronic Models of PD

One bottleneck associated with identifying therapeutic options for PD is the lack of a robust and pertinent animal model. While many models give potential results on a given aspect of parkinsonism, none fully recapitulate the pathognomonic lesions of PD (Dawson et al., 2010). Two broad categories of models are being used: neurotoxin-based (acute) and genetic-based (chronic) models. Neurotoxin models are the most popular. They can be produced by the use of the toxin 6-hydroxydopamine (6-OHDA), which preferentially kills DA neurons by production of free radicals (Przedborski et al., 1995), or 1-methyl-4-phenyl-1, 2, 3, 6-tetrahydropyridine (MPTP); Schober, 2004), which interferes with the mitochondrial metabolism, also producing free radicals (Petroske et al., 2001) and strong neuroinflammation (Luchtman et al., 2009, 2012). In addition to the loss of dopamine in the nigrostriatal DA system, they also reach extrastriatal regions, such as the subcortex and brainstem (Bezard et al., 2013). While we have learned much from toxin-induced PD models, their use in the development of disease-modifying therapies is challenging. A well-recognized caveat of toxin-induced PD models is that they mainly cause the degeneration of the nigrostriatal DA pathway, which induces robust motor symptoms, but poorly recapitulate symptoms related to most, but not all, pathways (Brown et al., 2012). These models, although very useful to test motor deficits or L-dopa responsiveness (Dawson et al., 2002), remain acute models where the progressive DA cell death is absent, and poorly mimic PD progression over time (Hattori and Sato, 2007). To circumvent these drawbacks many labs have opted for the creation of transgenic mice, widely based on *SNCA* and *LRRK2* mutants. However, transgenic *SNCA* mice, based on missenses mutations A30P, E46K or A53T, have led to limited parkinsonism (Deng and Yuan, 2014), especially in terms of nigrostriatal degeneration (Chesselet, 2008; Dawson et al., 2010). On the other side, the current cohort of *LRRK2* transgenic mice induce mild, if any, degeneration of nigrostriatal DA neurons, Lewy body formation, or behavior effects (Ramonet et al., 2011; Blesa and Przedborski, 2014). Of note though, *LRRK2* overexpression does accelerate the pathological consequences of *SNCA*^A53T^ in double transgenic mice (Lin et al., 2009). The latter study also suggests that the LRRK2 protein affects the intracellular trafficking and accumulation of SNCA protein. A transgenic rat overexpressing the G2019S mutation impaired dopamine uptake but did not show any nigral DA cell loss and striatal dopamine contents in aged rats (Zhou et al., 2011). Interestingly, transgene expression in adult animals using viral vectors can induce pronounced phenotypes in rodents, presumably by circumventing developmental compensatory effects, and by producing high level of transgene expression. In particular, vector-mediated expression of native or mutant SNCA can lead to DA neuron cell death and motor symptoms. Cognitive symptoms such as spatial learning and memory deficits (Hall et al., 2013), depression (Caudal et al., 2015) and emotional memory impairment, are influenced by VTA neurons (Alvarsson et al., 2016) in rats. In conclusion, animal models that recapitulate the early and late, motor and non-motor symptoms, within a time frame suitable to evaluate PD-modifying treatments, are still needed.

## Viral Vector-Mediated PD Effects in Nonhuman Primates

Nonhuman primates (NHPs) are particularly relevant in preclinical research because they share several genetic, physiological and anatomical similarities with humans. NHPs display complex cognitive functions, complex motor skills and a highly developed cerebral cortex (Verdier et al., 2015). Equally important, NHPs can be studied under controlled and humane experimental conditions. Interestingly, aged rhesus monkeys can naturally display a significant loss of tyrosine-hydroxylase and dopamine-transporter immunoreactivity correlated with motor impairments (Emborg et al., 1998). Furthermore, aged-related SNCA increase in rhesus monkeys has been observed in the nigral pathway (Chu and Kordower, 2007). These observations led to the idea that aged NHPs are at the threshold to develop a PD and, as a consequence, constitute a model of choice. Several viral vectors have been used to drive the development of PD in NHPs. Vector-mediated transgenesis is also versatile and transposable between species. Taking nothing away from the ground-breaking work performed in rodents, we believe that NHPs are needed to unravel PD induction, progression, therapeutic strategies (Emborg, 2007), and understand long-term pathophysiological, biochemical and behavioral anomalies. To develop these models, intracerebral injection of viral vectors bearing mutated *SNCA* or *LRRK2* have been tested for modeling “genetic” PD. Monkeys overexpressing simian or human SNCA coding for a protein with the A53T change *via* adeno-associated virus (AAV) vectors exhibit motor impairment and neuropathological features of PD including but not limited to: head position bias, loss of TH- and VMAT2-positive innervation throughout caudate nucleus and putamen, dystrophic neurites and swollen axons, SNCA-positive inclusions (Kirik et al., 2003). AAV vectors have been successfully used for expression of human SNCA^A53T^ in cynomolgus macaque SN, and led to a 50% loss of nigral DA neurons (Koprich et al., 2016). Lentivirus vectors expression of SNCA^A53T^ into the SN of rhesus monkeys resulted in more neuronal pathology and chronicity in monkey brains than in mouse brains (Yang et al., 2015). Of note, NHPs are also responsive to dopamine replacement therapies, and show complications resulting from long-term use such as dyskinesia and motor fluctuations when the medication is not working well.

In contrast to the small (~16 kDa) SNCA protein, the LRRK2 protein is ~250 kDa with at least seven different functional domains (Taymans and Greggio, 2016). The G2019S change located in the kinase domain, leads to a hyperkinase activity. LRRK2 was recently shown to be involved in the endoplasmic reticulum to Golgi export. Interestingly, this function is altered in the PD-related LRRK2^R1441C^ mutation located in the GTPase domain (Cho et al., 2014).

## Helper-Dependent Canine Adenovirus for Developing New PD Models

The *LRRK2* cDNA is about 8 kb and therefore a vector with an appropriately large cloning capacity is needed, and precludes its efficient expression in AAV and lentivirus vectors. A handful of attempts have been made to develop animal models expressing *LRRK2*^G2019S^ *via* viral vectors. To date, three vector platforms have been used to deliver the LRRK2^G2019S^ cDNA: human adenovirus type 5 (HAd5; Dusonchet et al., 2011; Tsika et al., 2014) were used in rats, herpes simplex virus (HSV) were used in mice. The HAd5-LRRK2^G2019S^ vector was injected into the striatum of rodents and due to its preferential transduction of glia cells and poor retrograde transport, the direct effect of LRRK2^G2019S^ on DA neurons in the nigra could not be addressed. Using a HSV vectors expressing LRRK2 or LRRK2^G2019S^, Lee et al. (2010) showed that the hyperkinase activity of LRRK2^G2019S^ was responsible for the PD phenotype, and that LRRK2 kinase inhibitors provide a potential neuroprotective treatment for PD. Interestingly, they also showed that overexpression of wild type LRRK2 caused neurite shortening *in vitro*.

**Figure 2:**
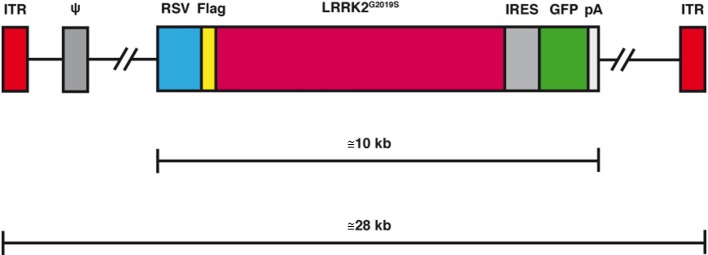
Schematic representation of the helper-dependent (HD) canine adenovirus-2 (CAV-2) vector expressing leucine-rich repeat kinase 2 (*LRRK2*)^G2019S^. While the HD genome is devoid of all viral coding sequences, it still retains the 200 bp inverted terminal repeat (ITR) at each end and the 150 bp packaging signal (ψ) at the left end of the genome. To create a stable capsid the genome must fill the interior of the capsid which therefore requires it to be 95%-105% of the 32 kbp wild type genome. Depending on the size of the expression cassette [here it contains the 600 bp Rous sarcoma virus early promoter (RSV), an internal ribosome entry signal (IRES), a green fluorescent protein cDNa 5GF] the *LRRK2* cDNA and a 250 polyA signal (pA), the remaining sequence is made up of noncoding intronic sequence from the human genome.

Clearly, the ability to efficiently and simultaneously deliver expression cassettes to multiple regions of the brain could be a notable plus for PD. Taking nothing away from the encouraging results when using HSV vectors (Goverdhana et al., 2005; Lee et al., 2010), we believe that we can improve PD modeling by using helper-dependent (HD) canine adenovirus (CAV-2; Junyent and Kremer, 2015; Figure 2). HD CAV-2 vectors have a unique combination of characteristics that make it ideally suited for PD modeling: CAV-2 vectors preferentially transduce neurons in rodent and NHPs, have no long-term impact on adult of newborn neuron homeostasis, have a 30 kb cloning capacity. Following injection in the rodent and NHP striatum, CAV-2 efficiently transits into afferent (axonal projections into the striatum) structures and is ≥100-fold more efficient than HAd5 vectors. This is particularly pertinent for PD modeling because efficient and stable gene transfer to DA neurons *via* injections into the SN is pernicious because DA neurons are particularly sensitive to stress (Albert et al., 2017). Due to the efficient retrograde transport of CAV-2 vectors in DA neurons (Soudais et al., 2001b; Schwarz et al., 2015), HD-LRRK2 vector can be delivered in the striatum, thus bypassing the potential damage incurred by SN injections.

HD CAV-2 vectors lead to long-term expression in the brain: transgene expression was stable for >1 year post-transduction (Soudais et al., 2001b) and cellular protein expression showed no change. These data demonstrate the low immunogenicity of HD CAV-2. Finally, scalable high titre production is also possible (Junyent and Kremer, 2015). We have invested significantly in optimizing CAV-2 vector cloning, creation and production parameters (Kremer et al., 2000; Soudais et al., 2001a; Fernandes et al., 2013, 2015a,b; Ibanes and Kremer, 2013), understanding CAV-2 uptake and trafficking (Soudais et al., 2000, 2001b; Chillon and Kremer, 2001; Martin-Touaux et al., 2002; Salinas et al., 2009), the physiological role of CAV-2’s receptor (Seiradake et al., 2006; Schoehn et al., 2008; Salinas et al., 2010; Rademacher et al., 2012; Piersanti et al., 2013; Kremer and Nemerow, 2015; Loustalot et al., 2015), and the *in vivo* use of the vectors [Junyent and Kremer, 2015 and del Rio et al. (in this Research Topics issue)]. HD CAV-2 vectors are therefore ideal for *LRRK2* cDNA delivery.

## New NHP Models of PD

### LRRK2^G2019S^ Expression in the Lemurian Primate *Microcebus murinus*

By complementing and extending the work performed in rodents, NHPs can help unravel PD induction, progression, therapeutic strategies (Emborg, 2007), and understand long-term pathophysiological, biochemical and behavioral anomalies. Although several type of NHPs are used to study neurodegenerative diseases (Verdier et al., 2015) the use of NHPs is time demanding, expensive, and the number of available animals for research is limited. To circumvent these issues, the *Microcebus murinus* (or gray mouse lemur) has the notable advantages to be small and efficiently bred in captivity. *M. murinus* are ideally for cerebral ageing and neurodegenerative studies because they develops complex behavioral (emotional), cognitive, and motor tests [(Joly et al., 2004, 2006, 2014; Picq, 2007; Trouche et al., 2010; Picq et al., 2012); for a review article, see also (Languille et al., 2012)], and also through transcriptomic studies (Abdel Rassoul et al., 2010) and transmissibility of neurodegenerative diseases (Mestre-Francés et al., 2012) or for gene transfer (Alba et al., 2012). In addition, its brain structure is similar to that of the humans, with a relative proportion of each region.

Recently, we generated HD CAV-2 vectors containing a LRRK2^G2019S^ expression cassette (HD-LRRK2^G2019S^) that we injected unilaterally into the putamen of *M. murinus*. We found preferential transduction of neurons at the injection site, and in numerous areas harboring neurons that project into the striatum. The long-term expression leads to the progressive unilateral loss of DA cells in the SNpc accounting for up to 30%–40% a decreased of the DA fibers, dystrophic neurites and swollen axons, characteristic of neurodegeneration. This neurodegeneration was accompanied by dopamine loss in the striatum, and PD-like motor symptoms (bradykinesia, rigidity, and difficulty in prehension; Mestre-Francés et al., 2018).

### *LRRK2*^G2019S^ Expression in Macaques

The promising outcomes obtained in the *M. murinus* model, prompted us to determine if CAV-2 vectors were also effective gene transfer tools in the *Macaca fascicularis* brain, and if CAV-2–mediated expression of LRRK2^G2019S^ in the SN neurons could induce pathological features associated with PD in a more complex NHP model. Our study demonstrated the neuronal tropism, retrograde transport, biodistribution, and efficacy of CAV-2 vectors expressing GFP in the *M. fascicularis* brain (Di Caudo et al., in preparation). Furthermore, we also demonstrated that CAV-2-mediated HD-LRRK2^G2019S^ expression in the SN leads to the loss of DA cells, neurite dystrophy, axon swelling and mitochondrial abnormalities. Unfortunately, animals injected with HD-LRRK2^G2019S^ into the striatum did not develop clear parkinsonian features, but they exhibited a significant reduction of striatal F-dopa uptake, indicating that they represent an early stage of the disease.

Together, these data demonstrate that robust PD NHP models can be generated using HD CAV-2 vectors and in turn could allow detailed evaluation of the therapeutic options for PD motor, emotional, and cognitive deficits.

## Viral Vectors for Developing Disease-Modifying Treatments

While currently available treatments can temporarily relieve the symptoms, they have little influence on the neurodegenerative process.

Neurotropic factors (NFs), which mediate pro-survival effects on neurons, potentially constitute a disease-modifying option. However, the results obtained with NFs are controversial, and largely depend on the model. For instance, in an AAV-SNCA-injected rat, the delivery of AAV-GDNF (glial cell line-derived neurotrophic factor), 2–3 weeks before AAV-SNCA injection, failed to demonstrate a neuroprotective effect (Decressac et al., 2012). In this case, *SNCA* overexpression resulted in Ret downregulation and disruption of GDNF signaling. However, a recent study demonstrated that Ret is not downregulated in PD patients (Su et al., 2017). In other studies, the therapeutic potential of NFs was demonstrated in toxin injected (6-OHDA and MPTP) rodents and NHPs, in which NFs reduced motor symptoms (Bilang-Bleuel et al., 1997; Kirik et al., 2000; Kordower et al., 2000, 2006; Eslamboli et al., 2003; Su et al., 2009). Following these encouraging results, clinical trials were conducted using AAV2-GDNF (still ongoing) or AAV2-neurturin (NRTN; Marks et al., 2010; Warren Olanow et al., 2015). Although the AAV2-NRTN trials demonstrated acceptable tolerance, after a 1-year follow-up, no significant improvement was observed in the “Unified PD Rating Scale” (UPDRS). However, *post hoc* analyses suggested that a subgroup of patients had beneficial effects (Marks et al., 2010). Post-mortem analysis of four patients showed that although surviving DA neurons were still present in the SN, very few co-stained with NRTN. These observations suggested that retrograde transport was inefficient (Bartus et al., 2011, 2015). Strikingly, these results were not predicted by the pre-clinical animal models used to establish the clinical protocol (MPTP-treated NHPs and 6-OHDA-injected rats) in which the surviving nigro-striatal DA neurons still had functional projections proficient for retrograde transport and could be efficiently rescued. In a subsequent study (Kordower et al., 2013), analysis of brains from untreated PD patients at different stages, showed that the putamen innervation had almost totally disappeared at 4 years post-diagnosis, whereas numerous DA neurons cell bodies were still present in the SNpc. Because most of the patients enrolled in the AAV2-NRTN trial were more than 5 years post-diagnosis, it is likely that their putaminal DA innervation had been lost or was dysfunctional. Although unsuccessful, the AAV2-NTRN trial was informative since it allowed: (i) to identify the limitations of the toxin-induced models; (ii) to suggest that disease-interfering treatments should be administered before disappearance the DA fibers; and (iii) supported enrolment of patients at earlier disease stages in gene therapy trials.

Therefore, disease-modifying treatments will need animal models that more faithfully recapitulate the mechanisms underlying the progression of PD, and should be administered at the earliest possible stage.

## Concluding Remarks

No animal model manifests all the characteristics of PD in humans, i.e., SNCA aggregation, DA reduction, progressive DA cell death, motor and non-motor symptoms. If transgenic models offer tremendous advantages over toxin-induced models, the overexpression of human disease causing mutated genes should be kept within the range of physiological levels. Viral vector-mediated local transgenesis offers the advantage to allow adjusting the transgene copy number to avoid confounding effects of a non-physiological overdosage. Clearly though it is difficult, if not impossible, to argue that NHP will not be the most informative path towards testing PD therapies. As underlined by Blesa and Przedborski (2014), *models are just models*, and should answer the question asked, not all the questions. Because NHPs have their own personality each animal can produce different emotional, motor, or cognitive behavior (that is why they are their own control). In addition, NHPs allow us to monitor early phases of the disease and follow-up. The combination of gene therapy and the use of NHPs should open new route to a disease-modifying treatment of PD.

## Data Availability

The datasets generated for this study are available on request to the corresponding author.

## Author Contributions

CL, M-RL, LT, EK, and J-MV wrote this review article. NM-F and GD edited and revised it.

## Conflict of Interest Statement

The authors declare that the research was conducted in the absence of any commercial or financial relationships that could be construed as a potential conflict of interest.
